# Supplementary material for: Usefulness of Serum as a Non-Invasive Sample for the Detection of Histoplasma capsulatum Infections: Retrospective Comparative Analysis of Different Diagnostic Techniques and Quantification of Host Biomarkers
Source: J Fungi (Basel). 2025 Jun 12;11(6):448. doi: 10.3390/jof11060448 (PMC12194025; doi:10.3390/jof11060448)
Supplement: Supplementary file 1 [file jof-11-00448-s001.zip › jof-3617735-supplementary.pdf]

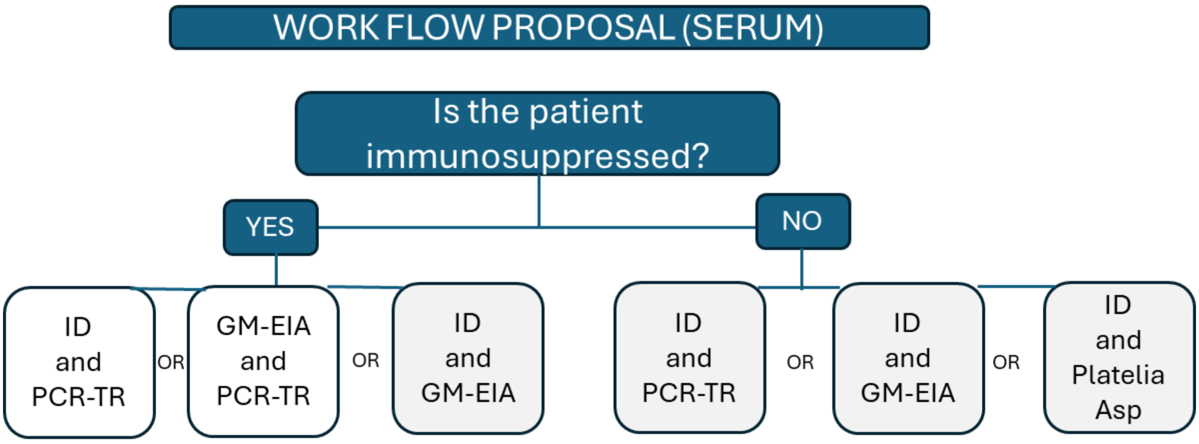

Supplementary Figure S1: Schematic workflow proposal to perform rapid diagnosis. In grey the combination of techniques with the highest sensitivity for that group of patients.
